# Saccharification Performances of Miscanthus at the Pilot and Miniaturized Assay Scales: Genotype and Year Variabilities According to the Biomass Composition

**DOI:** 10.3389/fpls.2017.00740

**Published:** 2017-05-29

**Authors:** Nassim Belmokhtar, Stéphanie Arnoult, Brigitte Chabbert, Jean-Paul Charpentier, Maryse Brancourt-Hulmel

**Affiliations:** ^1^AGPF, Institut National de la Recherche AgronomiqueOrléans, France; ^2^GCIE-Picardie, Institut National de la Recherche AgronomiqueEstrées-Mons, France; ^3^FARE Laboratory, Institut National de la Recherche Agronomique, Université de Reims Champagne-ArdenneReims, France; ^4^AgroImpact Unit, Institut National de la Recherche AgronomiqueEstrées-Mons, France

**Keywords:** Miscanthus, high-throughput pretreatment and saccharification, pilot-scale pretreatment and saccharification, hemicelluloses, cellulose, lignin, genotypic diversity, harvesting year

## Abstract

**HIGHLIGHTS**

- Biomass production and cell wall composition are differentially impacted by harvesting year and genotypes, influencing then cellulose conversion in miniaturized assay.
- Using a high-throughput miniaturized and semi-automated method for performing the pretreatment and saccharification steps at laboratory scale allows for the assessment of these factors on the biomass potential for producing bioethanol before moving to the industrial scale.

The large genetic diversity of the perennial grass miscanthus makes it suitable for producing cellulosic ethanol in biorefineries. The saccharification potential and year variability of five genotypes belonging to *Miscanthus* × *giganteus* and *Miscanthus sinensis* were explored using a miniaturized and semi-automated method, allowing the application of a hot water treatment followed by an enzymatic hydrolysis. The studied genotypes highlighted distinct cellulose conversion yields due to their distinct cell wall compositions. An inter-year comparison revealed significant variations in the biomass productivity and cell wall compositions. Compared to the recalcitrant genotypes, more digestible genotypes contained higher amounts of hemicellulosic carbohydrates and lower amounts of cellulose and lignin. In contrast to hemicellulosic carbohydrates, the relationships analysis between the biomass traits and cellulose conversion clearly showed the same negative effect of cellulose and lignin on cellulose digestion. The miniaturized and semi-automated method we developed was usable at the laboratory scale and was reliable for mimicking the saccharification at the pilot scale using a steam explosion pretreatment and enzymatic hydrolysis. Therefore, this miniaturized method will allow the reliable screening of many genotypes for saccharification potential. These findings provide valuable information and tools for breeders to create genotypes combining high yield, suitable biomass composition, and high saccharification yields.

## Introduction

To meet targeted demands for alternative fuel sources with less environmental impact, a large set of lignocellulosic biomasses has been explored within the last two decades (Wyman, 1994; Rubin, 2008). One of the most promising feedstocks is the perennial grass miscanthus due to its high biomass production per hectare with a low environmental impact (Clifton-brown et al., 2004; Lewandowski and Schmidt, 2006; Hastings et al., 2008; Cadoux et al., 2014) and its minor impact on the food supply (Clifton-brown et al., 2004; Heaton et al., 2008; Brosse et al., 2012). The genus *Miscanthus* comprises 20 species (Hodkinson et al., 2002), including *Miscanthus* × *giganteus, Miscanthus sinensis, Miscanthus sacchariflorus* and *Miscanthus tinctorius*, which are characterized by a high yield and energy content (Cadoux et al., 2014; Lee and Kuan, 2015). The mostly studied genotype in Europe and North America is *M*. × *giganteus*, which is a sterile interspecific hybrid of *M. sacchariflorus* and *M. sinensis* (Brosse et al., 2012).

The efficient hydrolysis of cellulose into fermentable glucose using fungal cellulolytic enzymes is a key step in the biorefinery process (Himmel et al., 2007). However, to provide rigidity, structural support, and protection against pathogens in living plants, the cellulosic fraction is part of a cohesive network of phenolic, non-cellulosic polysaccharides, and protein polymers (Pauly and Keegstra, 2008; De Souza et al., 2015). The inhibitory effect of cellulose crystallinity on cellulases and the non-productive binding of enzymes to lignin have been demonstrated (Yang and Wyman, 2004; Berlin et al., 2006). Furthermore, monocots, such as miscanthus, are characterized by the presence of hydoxycinnamic acids such as *p*-coumaric acids and ferulic acids, which play a significant role in cross-linking arabinoxylans to lignin hindering then cellulose digestibility (Iiyama et al., 1990; Ralph, 2010). In addition, non-cellulosic components have been reported to differ in their distribution, structure, and extractability across different harvests, organs, and miscanthus genotypes (Le Ngoc Huyen et al., 2010; Xue et al., 2013; Costa et al., 2016). Variability in cellulose and lignin content has been highlighted between miscanthus harvests in stems while glycans matrix was more easily extractable in leaves using profiling of polysaccharides epitopes pattern. Total contribution of leaves to total biomass seems to be a determining factor for saccharification efficiency (Costa et al., 2016). Moreover, pectins and mannans impact was established when lignin did not impact saccharification (De Souza et al., 2015). All these findings confirmed how complex is the understanding of cell wall recalcitrance to deconstruction.

To overcome these structural limitations, the optimal digestion of a lignocellulosic network needs physical and/or chemical pretreatments (Yang and Wyman, 2008; Hendriks and Zeeman, 2009). Several pretreatment technologies have been extensively explored during the last decades, and their effectiveness was demonstrated by disrupting the lignocellulose structure of different energy crops (Mosier et al., 2005; Wyman et al., 2005). Steam explosion is one of the most promising pretreatments and has been successfully applied to miscanthus by solubilizing hemicelluloses and disrupting cellulose fibers during rapid depressurization, thereby increasing cellulose digestibility (Sørensen et al., 2008; Yeh et al., 2016). Hot water treatment has also been shown to be very efficient in increasing cellulose conversion of miscanthus (Li H.-Q. et al., 2013). To realize a techno-economic evaluation of the bioethanol production process, acid hydrolysis, wet oxidation, and steam explosion have been extensively tested and validated at the pilot scale on different biomasses, such as wheat straw and corn stover (Schell et al., 2003, 2004; Thomsen et al., 2006; Jørgensen et al., 2007).

In addition to the achievement of efficient pretreatments for second-generation bioethanol, the comparison of the saccharification potentials of a large set of biomass requires efficient and rapid tests including physico-chemical pretreatments. Some miniaturized and automated methods have been developed to evaluate large biomass populations. These tools are based on performing pretreatment and saccharification assays in 96-well microplate systems (Gomez et al., 2010; Santoro et al., 2010; Selig et al., 2010; Studer et al., 2011). Methods developed by Gomez et al. (2010, 2011) are based on using 96-well PCR plates thus allowing only low or moderate heating during pretreatment step. Those described by Studer et al. (2011) and Selig et al. (2010) are characterized by using metallic reactors that allow high temperature and pressure pretreatments in addition to resistance to corrosion and catalysts, such as dilute sulfuric acid and sodium hydroxide. The same team reported improvements of NREL's method, notably the replacing of aluminum by Hastelloy for 96-well plate, the manufacturing and use of polytetrafluoroethylene film tape for plate sealing rather than aluminum foil tape (Biswal et al., 2015; Healey et al., 2016; Serba et al., 2016). These promising methods make breeding and the best selection of less recalcitrant feedstocks for cellulosic ethanol possible.

Biomass production and cell wall composition have been shown to be highly variable among *Miscanthus* genotypes (Allison et al., 2011; Arnoult and Brancourt-Hulmel, 2015; Arnoult et al., 2015a; Costa et al., 2016). Moreover, the miscanthus biomass production and composition may be impacted by the environment, particularly by climatic conditions that can differ with harvesting date and year (Le Ngoc Huyen et al., 2010; Arnoult et al., 2015a,b). This potentially allows breeding of miscanthus varieties that adapt to their environment, resulting in a high yield, a suitable biomass composition, a high saccharification potential, and bioethanol conversion (Hodgson et al., 2010, 2011; Lygin et al., 2011).

The main objective of our study, therefore, is to explore the saccharification potential of miscanthus genotypes and the year variability effect by assessing the saccharification performances.

Our first hypothesis was that the saccharification potential was variable according to genotypes and harvesting years due to variations in biomass production and biomass composition. Therefore, we developed a high-throughput miniaturized and semi-automated method to evaluate the saccharification potential, and (i) for several years, we used this method to determine the impact of the harvesting year on the enzymatic digestibility of 5 miscanthus genotypes belonging to the *M*. × *giganteus* and *M. sinensis* species. Additionally, we examined the effect of the harvesting year on the biomass production and composition of these 5 genotypes. (ii) In light of these results, we investigated the relationships between biomass production, cell wall composition, and cellulose conversion according to the harvesting year. We studied five miscanthus genotypes harvested at the end of the winter in the fourth, fifth, and seventh years of cultivation.

Our second hypothesis was that the use of a satisfactory and relevant method at the miniaturized scale using a minimum amount of matter was reliable enough to assess the performance at the pilot scale. To address this hypothesis, we explored, at the pilot scale, the saccharification of the previously harvested 5 genotypes at the end of the winter of year 7. Then, we correlated the results of the cellulose conversion yields obtained between the pilot scale and the high-throughput miniaturized and semi-automated method we developed.

This study will provide useful knowledge and tools to breed suitable *Miscanthus* genotypes for cellulosic ethanol production.

## Materials and methods

### Location of the experimental field

The experimental field is located in the Hauts-de-France region of Northern France (49°53 N, 3°00 E) at the INRA experimental unit in Estrées-Mons.

### Management of the trial

The trial was planted by hand in spring 2007 at a rhizome planting density of 2 plants per m^2^. The plants were watered immediately after planting to ensure good root contact with the soil. No irrigation was applied during the following years of cultivation. No fertilizer was applied. The weeds were controlled each year by hand and machine hoeing.

### Field production of the *Miscanthus* genotypes

Twenty-one *Miscanthus* genotypes were planted in 2007 in a randomized complete block experimental design with three blocks (for details, see Arnoult et al., 2015a). Among these 21 genotypes, the following five genotypes were studied: two genotypes were identified as *M. sinensis* (ROT and SIL), and three genotypes were identified as *M*. × *giganteus* (FLO, GIB, and H8). Among the three *M*. × *giganteus* genotypes, H8 was considered an *M*. × *giganteus* genotype as it was a hybrid between *M. sacchariflorus* and *M. sinensis*. Using Amplified Fragment Length Polymorphism (AFLP) markers, the genotype named “FLO” belonged to the *M*. × *giganteus* species (Rambaud, personal communication). These five genotypes were chosen among the 21 previously studied genotypes (Arnoult et al., 2015a) due to (i) their relatively high biomass production per hectare and (ii) their contrasted biomass composition, particularly for cellulose and lignin contents.

These five genotypes were harvested from the experimental field at the end of the winter of years 4, 5, and 7 (2011, 2012, and 2014, respectively). A surface of 16 m^2^ was cut 5 cm above the ground using a reed harvester.

Two sets of samples were collected for each of the 5 genotypes as follows: (i) a sample of 70 kg of biomass harvested in the whole 3 blocks in year 7 was used for the pilot-scale assay, and (ii) a sample of ~500 g of biomass randomly chosen from the biomass harvested on each of the 3 blocks of the trial in years 4, 5, and 7 was used for the high-throughput assays and cell wall components quantification. For the pilot-scale assay, (i) we harvested 7th year miscanthus in 2014, which corresponds to the first year when the pilot was operational, and (ii) we needed to pool the biomass from three blocks of the trial to provide enough biomass for the pilot assays (70 kg).

### Pilot-scale assays

#### Biomass preparation and steam explosion pretreatment

For each of the five genotypes, the 70 kg of biomass harvested from the experimental field was carried on the pilot site (*Procethol 2G*, Pomacle, France) and stored until its treatment in a dedicated storage area sheltered from rain. Then, all the matter of each genotype was separately ground in the pilot and resulted in a biomass size between 20 and 100 mm.

Thereafter, the ground biomass was loaded in 1 m^3^ containers in the presence of sulfuric acid. After pressing, the presoaked biomass was placed in a steam reactor and heated. Moving the biomass into an atmospheric chamber caused a quick depressurization, resulting in the explosion of the miscanthus fibers. Only one replicate of this step was performed due to the limitation in available biomass amounts in the field. Additional measures on 21 samples from the same plot of *M*. × *giganteus* showed a good accuracy of the results at pilot scale (coefficient of variation = 1.99%, confidential data, Procethol 2G).

#### Enzymatic hydrolysis of steam-exploded miscanthus and quantification of released glucose

A standard test of enzymatic hydrolysis was performed on the pretreated biomass. The pretreated biomass was incubated for 72 h with an enzymatic solution containing 10 IU of enzymes.g^−1^ DM. The enzymatic solution contained Genencor GC220 cellulase and β-glucosidase (N188, Novozymes). This step was replicated twice.

Released glucose after the pretreatment and enzymatic hydrolysis was performed by a high-performance anion-exchange chromatography (HPAEC) as described previously (Le Ngoc Huyen et al., 2010). This quantification of glucose was replicated twice.

### Cell wall components quantification (LANO laboratory)

#### Sample preparation

For each of the 5 genotypes harvested from three individual blocks of the trial, the sample of ~500 g of the biomass harvested was dried at 65°C for 4 days in a well-ventilated oven. These dried samples were ground with a crusher (Viking, model GE 220, France) to a coarse size and then ground with a hammer crusher (Gondard Productions model, France) to pass through a 1-mm screen, as recommended for subsequent fiber analysis by Van Soest and Wine (1967).

#### Determination of the cellulose, hemicellulosic carbohydrates, and lignin contents

The previous samples were analyzed by the laboratory LANO (Saint-Lô, France) for neutral detergent fiber (NDF), acid detergent fiber (ADF), and acid detergent lignin (ADL), according to a protocol that was adapted from the Van Soest method (Van Soest and Wine, 1967). Briefly, the NDF fraction corresponded to the ash-corrected residue that remained after refluxing for 60 min in a neutral-buffered detergent solution. The ADF fraction corresponded to the ash-corrected residue remaining after refluxing the samples in a solution of hexadecyltrimethylammonium bromide in 0.5 mol/L sulfuric acid. The ADL fraction was obtained by treating ADF with 72% sulfuric acid.

The NDF is considered to represent cellulose, hemicellulosic carbohydrates, and lignin; the ADF consists of cellulose and lignin; and the ADL consists of lignin (Van Soest and Wine, 1967). The cellulose, hemicellulosic carbohydrates (hemicelluloses), and lignin contents of each sample were estimated by subtracting the corresponding values from the NDF, ADF, and ADL fractions as shown below in Equations (1–3):

(1) $$\begin{array}{l} \text{Cellulose content}=\text{ADF}-\text{ADL} \end{array}$$

(2) $$\begin{array}{l} \text{Hemicellulosic carbohydrates content}=\text{NDF}-\text{ADF} \end{array}$$

(3) $$\begin{array}{l} \text{Lignin content}=\text{ADL} \end{array}$$

The analytical dry matter content of each sample was determined at 103°C to express all the previous values in percentage of dry matter (% DM).

The Neutral Detergent Soluble (NDS) corresponds to the extractives and was calculated as following:

$$\begin{array}{l} \text{NDS}=100-\text{NDF} \end{array}$$

We calculated several ratios, the lignocellulose index (LCI) among others, which corresponded to the ratio between lignin and (lignin + cellulose + hemicellulosic carbohydrates).

### Performing the high-throughput assays

#### High-throughput pretreatment and saccharification

All samples were dried overnight at 50°C prior to ball milling to 1 mm screen in a fully automated system designed and provided by *Labman*® to French National Institute for Agricultural Research, Orleans. Biomass was then dispensed into 96-well microplates using another automatic dispenser station *Labman*®. Each sample was dispensed into 4 individual wells as a technical repetition.

For the hot water pretreatment, we used Hastelloy 96-well SBS-type microplates manufactured by Aspen machining® (*Golden*, Co, USA) based on drawings generously provided by NREL (*Golden*, CO, USA). After dispensing precisely 5 mg of biomass per well, 300 μL of deionized water were added to each sample. Hastelloy microplates were then tightly sealed with an adhesive film (3M®, 5,490) and clamped in the loader to be introduced in a 2-gallon stainless *Parr* reactor (4,665). Hot water pretreatment was performed at 180°C for 40 min. Heating was carried out using wet steam generated by an E-3000 Steam Generator provided by *Cellkraft*® (Sweden). This steam was heated at 180°C and introduced in the top of the Parr reactor. Pretreatment was stopped by depressurizing *Parr* vessel and cooling by adding water through valves placed in the top of the reactor.

After centrifugation (5,800 g, 10 min, 4°C), the adhesive film was removed, and 40 μL of the enzymatic solution containing 91 IU of cellulases.g^−1^ of DM in sodium acetate buffer (1 M, pH 5) were added based on NREL assays (Selig et al., 2010). Microplates were then sealed again using an adhesive film and incubated at 50°C during 70 h without shaking. For each sample, a control well was carried out without enzymes in order to estimate released non-cellulosic carbohydrates.

#### Quantification of released glucose in microplates

Released glucose, after the pretreatment and enzymatic hydrolysis, was collected after centrifuging the microplates and quantified using the GOPOD assay kit (*Megazyme*®, USA). Glucose released in control wells was subtracted to determine the cellulose conversion yields expressed as a percentage of the cellulose content.

### Data analysis

Using data from the high-throughput assays, we calculated the biomass composition from the mean of three technical replicates, while the saccharification results were obtained from the mean of four technical replicates for each genotype. All sample CVs were <10%. Samples with CVs >10% were removed.

RStudio (version 3.3.2) was also used to perform an analysis of variance (ANOVA) to assess the effect of the harvesting year and genotype on the biomass production, cell wall composition, and saccharification yields. We also used the Tukey–Kramer test to achieve multiple comparison tests.

For these analyses, two linear models were built;

- A first model taking into account the block factor to test its effect on the studied variables as following:
  $$\begin{array}{l} Y_{\text{ijk}}=\mu +\alpha_{\text{i}}+\beta_{\text{j}}+\gamma_{\text{k}}+{\left(\alpha \beta \right)}_{\text{ij}}+{\left(\alpha \gamma \right)}_{\text{ik}}+\varepsilon_{\text{ijk}} \end{array}$$
  where Y_ijk_ is the phenotypic value of clone *i* in block *j* during the harvesting year *k*; μ is the overall mean; α_i_ is the fixed effect of the clone *i*; β_j_ is the fixed effect of the block *j*; γ_k_ is the fixed effect of the harvesting year *k*; (αβ)_ij_ is the fixed interaction between clone *i* and the block *j*; (αγ)_ik_ is the fixed interaction between clone *i* and the harvesting year *k*; and ε_ijk_ is the residual error for clone *i* for block *j* during the harvesting year *k*.
- As the block effect was not significant for all the variables tested (data not shown), we used in the following (ii) a second model as follows:
  $$\begin{array}{l} Y_{\text{ij}}=\mu +\alpha_{\text{i}}+\gamma_{\text{j}}+{\left(\alpha \gamma \right)}_{\text{ij}}+\varepsilon_{\text{ij}} \end{array}$$
  where Y_ij_ is the phenotypic value of clone *i* during the harvesting year *j*; μ is the overall mean; α_i_ is the fixed effect of the clone *i*; γ_j_ is the fixed effect of the harvesting year *j*; (αγ)_ij_ is the fixed interaction between clone *i* and the harvesting year *j*; and ε_ij_ is the residual error for clone *i* during the harvesting year *j*.

## Results

### Impact of harvesting year according to genotype

#### Biomass production

**Figure 1:**
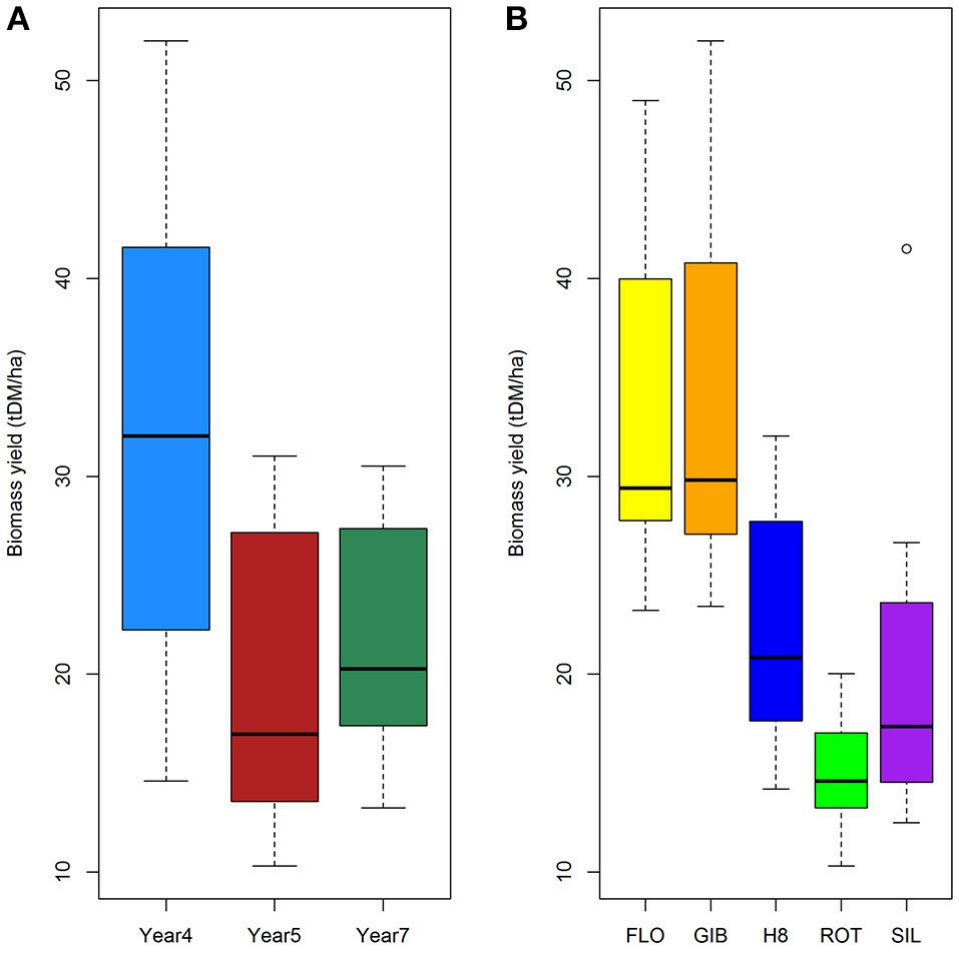
**Impact of harvesting year (A)** and genotypes **(B)** on biomass production.

The results summarized in Figure 1 and the analysis of variance (Table 1) clearly indicated that the biomass production is significantly impacted by the harvesting year (*F* = 39.8, *p* < 0.001) and genotype (*F* = 32.5, *p* < 0.001) with a significant interaction between both factors (*F* = 2.6, *p* < 0.05). Indeed, the biomass production reached the highest yields at year 4 for the FLO and GIB genotypes, which reached 45.43 and 43.54 tDM.ha^−1^, respectively, while the lowest yields were obtained at year 7, when the genotype H8, ROT, and SIL yields ranged from 11 to 19 tDM.ha^−1^. Furthermore, multiple Tukey–Kramer comparison highlighted that the miscanthus genotypes were divided into the following two sub-populations: GIB-FLO and H8-ROT-SIL (Supplementary Image 1).

**Table 1:** **Analysis of variance of traits related to biomass production and chemical composition of miscanthus genotypes**.

		***F*-value**	***p*-value**
Biomass yield	Genotype	32.5	<0.001
	Harvesting year	39.8	<0.001
	Genotype: Year	2.6	<0.05
NDF	Genotype	31.9	<0.001
	Harvesting year	16.8	<0.001
	Genotype: Year	2.3	<0.05
Extractives	Genotype	35.5	<0.001
	Harvesting year	16.9	<0.001
	Genotype: Year	2.3	<0.05
Cellulose	Genotype	263.6	<0.001
	Harvesting year	6.4	<0.01
	Genotype: Year	3.3	<0.01
Hemicellulosic carbohydrates	Genotype	124.5	<0.001
	Harvesting year	77.0	<0.001
	Genotype: Year	3.8	<0.01
Lignin	Genotype	42.9	<0.001
	Harvesting year	40.4	<0.001
	Genotype: Year	1.2	>0.05
Hemicellulosic carbohydrates/Cellulose ratio	Genotype	214.5	<0.001
	Harvesting year	63.7	<0.001
	Genotype: Year	3.4	<0.01
Lignin/Cellulose ratio	Genotype	19.7	<0.001
	Harvesting year	39.0	<0.001
	Genotype: Year	0.9	>0.05
LCI	Genotype	36.7	<0.001
	Harvesting year	38.2	<0.001
	Genotype: Year	0.9	>0.05
Released glucose from untreated biomass	Genotype	19.0	<0.001
	Harvesting year	47.9	<0.001
	Genotype: Year	6.9	<0.001
Released glucose from hot water-treated biomass	Genotype	109.5	<0.001
	Harvesting year	50.1	<0.001
	Genotype: Year	2.2	<0.05

#### Biomass composition

**Figure 2:**
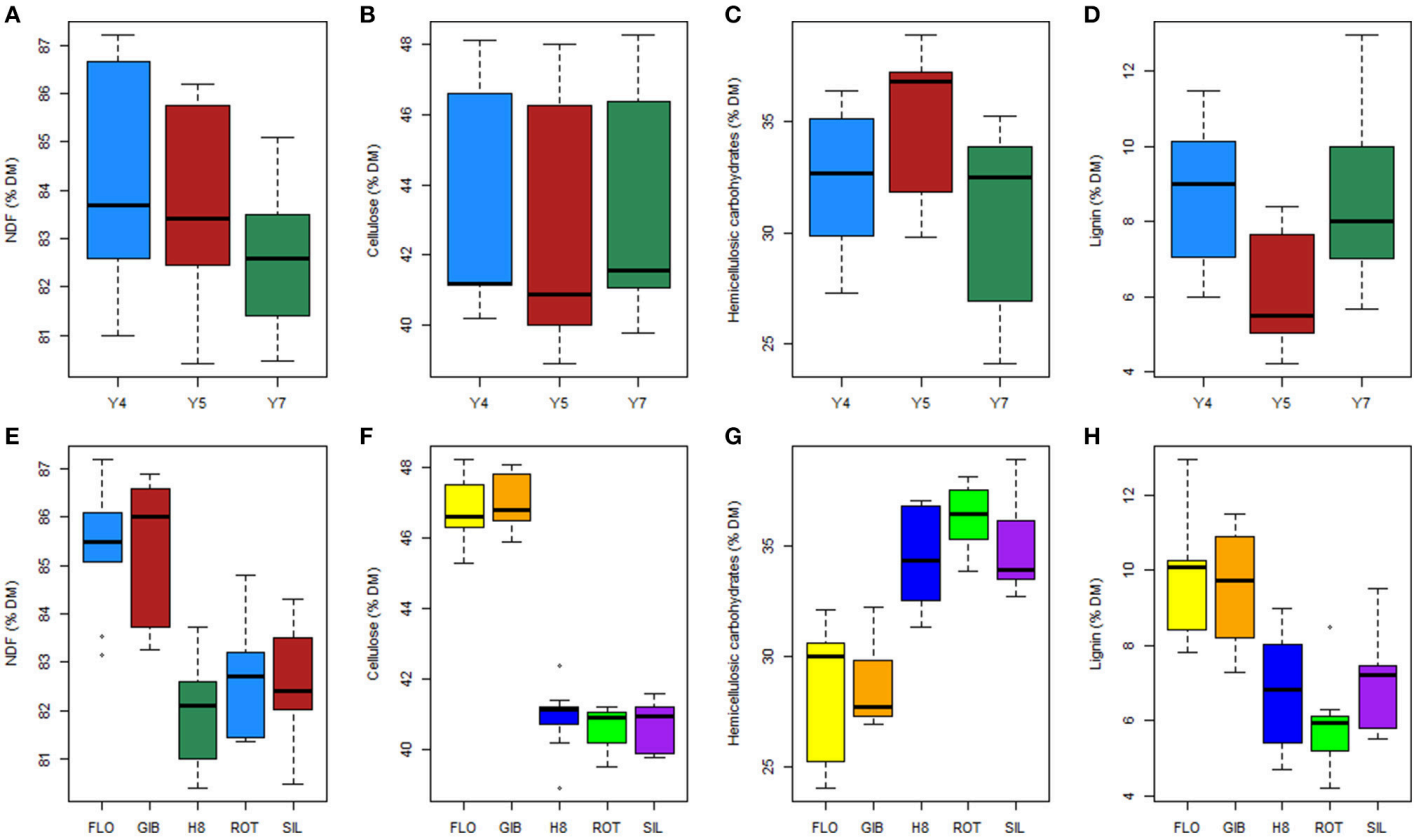
**Effect of harvesting year (A–D)** and genotypes **(E–H)** on the biomass composition.

The cell wall fraction as estimated by NDF ranged from 80 to 87% of the biomass dry matter; NDS referred hereinafter as extractives, ranged 12.9–19.0, 13.8–19.5, and 14.9–19.5% of the biomass dry matter at years 4, 5, and 7, respectively. The determination of the harvesting year and genotype effects on dry matter and cellulose content was performed using an ANOVA and showed significant effects and interaction between these two factors (Table 1). However, the NDF and cellulose content seemed less affected by the harvesting year (*F* = 16.8 and *p* < 0.001, *F* = 6.4 and *p* < 0.01, respectively) than the genotype (*F* = 31.9 and *p* < 0.001, *F* = 263.6 and *p* < 0.001, respectively). Multiple comparisons also highlighted the following two sub-populations: GIB-FLO and H8-ROT-SIL (Supplementary Image 1). The NDF and cellulose contents are summarized in Figures 2A,B,E,F.

The same approach revealed that the content in hemicellulosic carbohydrates was also impacted by the harvesting year and genotype with a stronger effect of the latter (*F* = 124.5 and *p* < 0.001) compared to the former (*F* = 77 and *p* < 0.001). Our results also indicated a significant interaction between these two factors (*F* = 3.8 and *p* < 0.01). Overall, hemicellulosic carbohydrates content appeared to be higher in year 5 and lower in year 7 (Figure 2C). Additionally, Tukey–Kramer comparison confirmed that the two sub-populations of genotypes GIB-FLO contained less than 30% of hemicellulosic carbohydrates, while the second one, composed of H8, ROT, and SIL, reached more than 35% (Figure 2G).

Exploring the lignin content revealed significant and strong effects of harvesting year and genotype but no interaction between these two factors (Table 1). The results summarized in Figure 2D indicated that the lignin content was higher at year 4 and lower at year 5. Two sub-populations of genotypes were also found for this trait. In contrast to the H8, ROT, and SIL group, with <7% of DM (Figure 2H), the GIB-FLO group showed the highest lignin amounts (~10% of DM).

**Figure 3:**
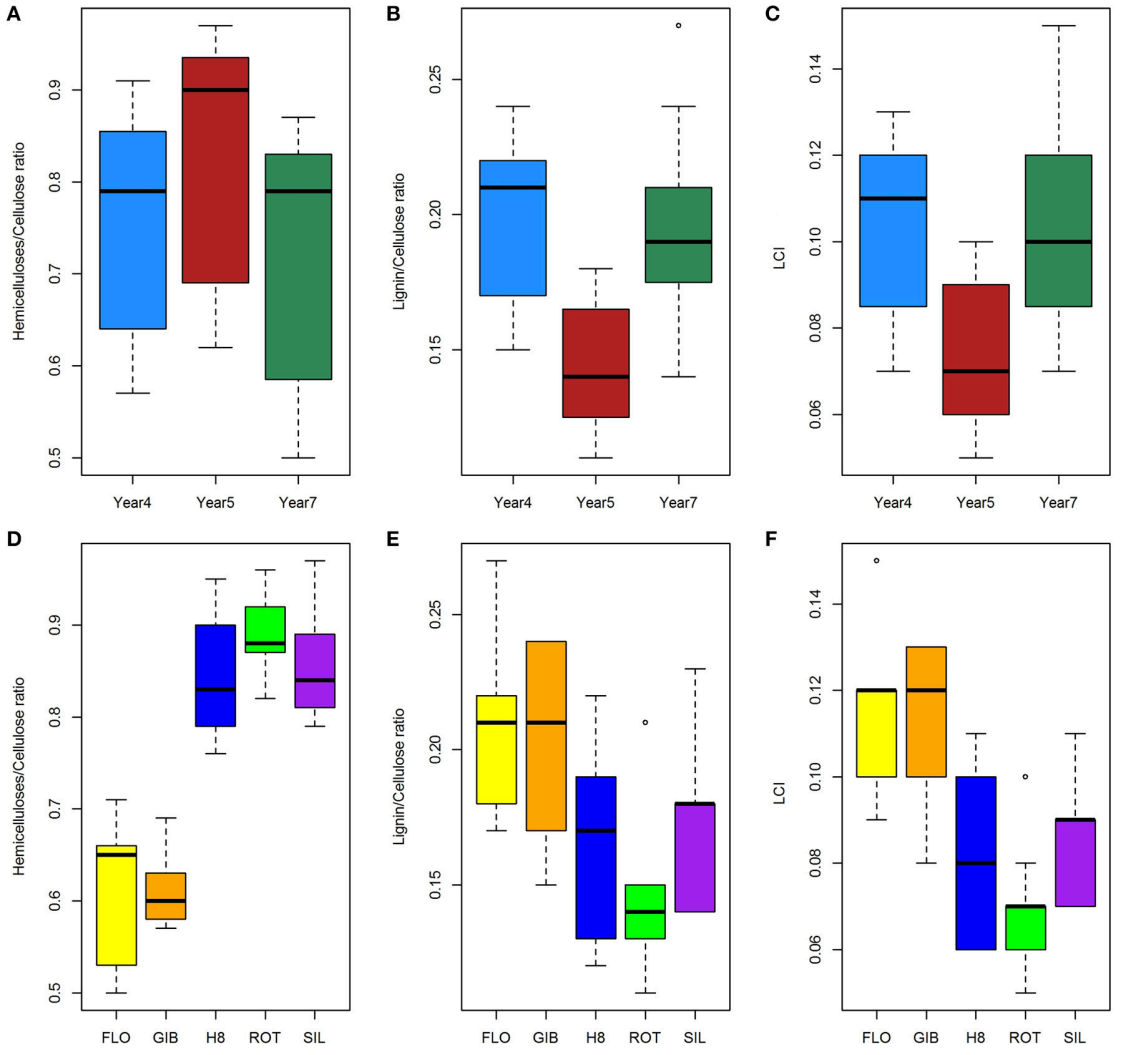
**Effect of harvesting year (A–C)** and genotypes **(D–F)** on Hemicellulosic carbohydrates/Cellulose, Lignin/Cellulose, and LCI ratios.

Variance analysis was also applied to the Hemicellulosic carbohydrates/Cellulose ratio, Lignin/Cellulose ratio, and LCI. The results summarized in Table 1 indicate that all these traits were significantly affected by harvesting year and genotype, but an interaction between these factors was only found for the Hemicellulosic carbohydrates/Cellulose ratio. Specifically, the Hemicellulosic carbohydrates/Cellulose ratio was highest at year 5, which is in contrast to the Lignin/Cellulose ratio and LCI, which were the lowest the same year (Figures 3A–C). Based on the genotype, the group formed by H8, ROT, and SIL expressed the highest Hemicellulosic carbohydrates/Cellulose ratio, while the other group highlighted the highest Lignin/Cellulose ratio and LCI (Figures 3D–F).

#### Enzymatic hydrolysis of untreated and hot water-treated genotypes

Quantification of released glucose after enzymatic hydrolysis of untreated and hot water- treated miscanthus highlighted a very strong and significant effect of harvesting year and the genotypes according to analysis of variance (Table 1). Additionally, examination of the interaction between genotype and harvesting year indicated the significant interaction for the untreated samples (*F* = 6.9 and *p* < 0.001).

**Figure 4:**
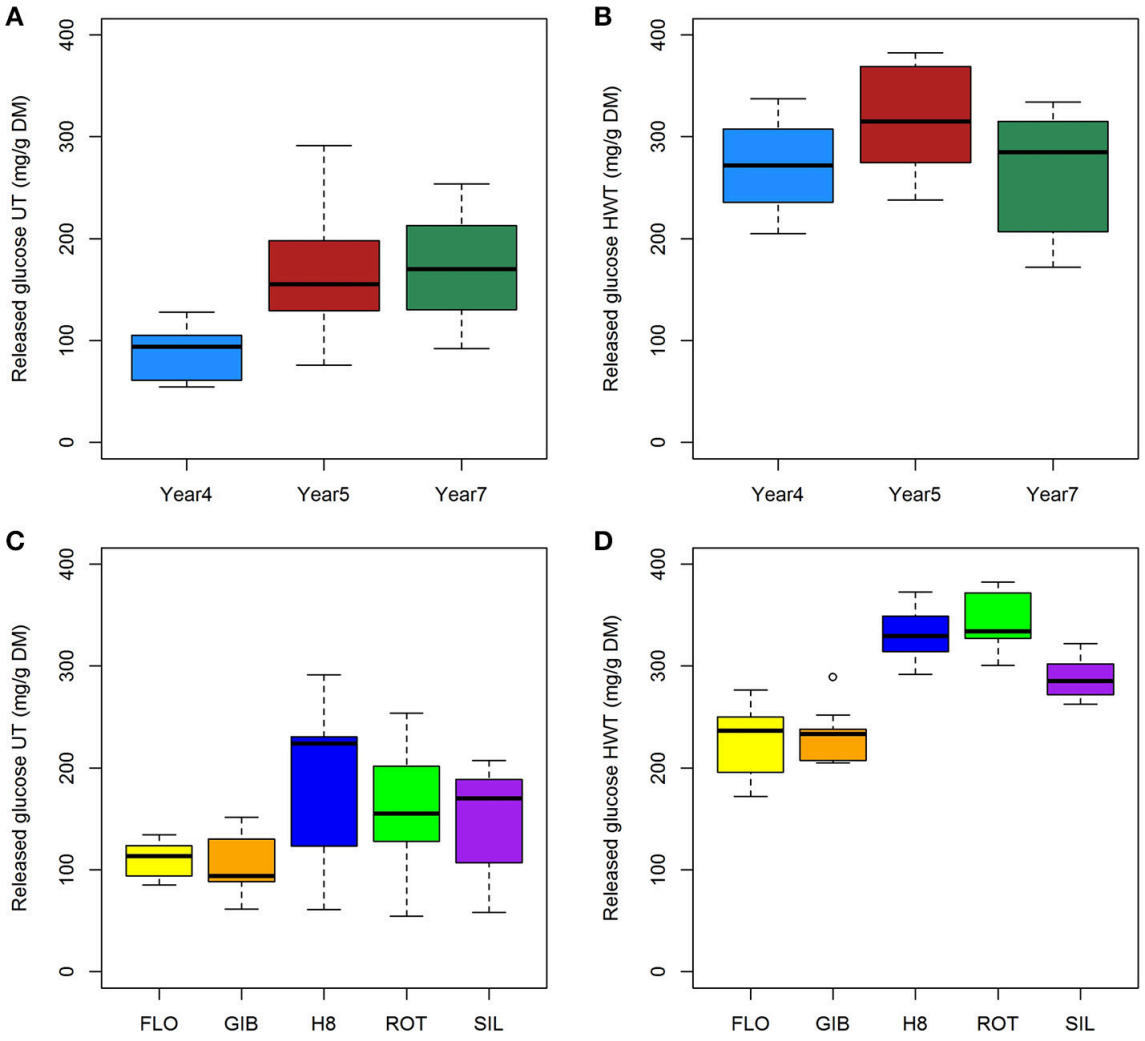
**Impact of harvesting year (A,B)** and genotypes **(C,D)** on released glucose content from untreated **(A,C)** and hot water-treated **(B,D)** miscanthus.

The ANOVA results clearly indicated that the harvesting-year effect on saccharification was more important without pretreatment, while genotype showed a much higher impact after the hot water treatment (Table 1). These findings can also be observed in Figure 4 where, compared to year 4, the highest release of glucose was observed at years 5 and 7 (Figures 4A,B). After the hot water pretreatment, the impact of harvesting year was limited to year 5; while, compared to FLO and GIB, the genotype group composed of H8, ROT, and SIL appeared to be more digested, reaching more than 300 mg/g DM of released glucose (Figures 4C,D).

### Relationships between biomass production, content of main cell-wall components, and cellulose conversion

Investigation of the relationships between miscanthus traits and released glucose after enzymatic hydrolysis of untreated and hot water-treated samples was conducted by focusing on the effects of genotype and harvesting year.

Correlation between enzymatic hydrolysis efficiency and cellulose, hemicellulosic carbohydrates, and lignin contents was significantly affected by the genotype. Indeed, the results detailed in Table 2 indicate that saccharification of the untreated sample was slightly affected by extractives content (*r* = 0.52) and Biomass yield (*r* = −0.47) in FLO-GIB group while the group H8-ROT-SIL is mainly negatively impacted by lignin content (*r* = −0.55), Lignin/Cellulose (*r* = −0.59), and LCI (*r* = −0.52) ratios. Glucose release from hot water-treated samples belonging to FLO and GIB genotypes was strongly impacted by hemicellulosic carbohydrates content (*r* = 0.96) and Hemicellulosic Carbohydrates/Cellulose ratio (*r* = 0.95). This group is also negatively impacted by extractives (*r* = −0.53), cellulose (*r* = −0.52), and lignin (*r* = −0.78) contents. However, glucose released from pretreated genotypes H8, ROT, and SIL was less impacted by the cell wall composition and biomass production (Table 2).

**Table 2:** **Relationship analysis between biomass traits and enzymatic hydrolysis of cellulose based on correlation analysis (***r***-values)**.

		**Genotype**		**Harvesting year**		
		**FLO, GIB**	**H8, ROT, and SIL**	**Year 4**	**Year 5**	**Year 7**
Untreated biomass	Extractives	+0.52	+0.39	+0.05	+0.34	+0.57
	Cellulose	+0.03	−0.22	−0.03	−0.74	−0.78
	Hemicellulosic carbohydrates	−0.35	+0.23	+0.23	+0.63	+0.81
	Lignin	+0.08	−0.55	−0.41	−0.67	−0.83
	Biomass yield	−0.47	−0.35	+0.00	−0.53	−0.59
	Hemicellulosic carbohydrates/Cellulose ratio	−0.31	+0.25	+0.16	+0.69	+0.81
	Lignin/Cellulose ratio	+0.09	−0.59	−0.55	−0.63	−0.81
	LCI	+0.17	−0.52	−0.46	−0.61	−0.85
Hot water-treated biomass	Extractives	−0.53	−0.04	+0.74	+0.84	+0.73
	Cellulose	−0.52	−0.41	−0.88	−0.89	−0.92
	Hemicellulosic carbohydrates	+0.96	+0.59	+0.82	+0.89	+0.94
	Lignin	−0.78	−0.62	−0.74	−0.93	−0.91
	Biomass yield	+0.04	−0.51	−0.88	−0.77	−0.74
	Hemicellulosic carbohydrates/Cellulose ratio	+0.95	+0.60	+0.87	+0.89	+0.94
	Lignin/Cellulose ratio	−0.74	−0.61	−0.58	−0.91	−0.86
	LCI	−0.79	−0.64	−0.72	−0.92	−0.90

Correlation analysis, based on harvesting year, has also highlighted the differential impact of biomass traits on enzymatic hydrolysis of untreated and pretreated miscanthus genotypes. Those samples harvested at year 4 and hot water-treated were strongly affected by cellulose (*r* = −0.88) and hemicellulosic carbohydrates (*r* = 0.82) contents, followed by slight effect of lignin content (*r* = −0.74). In contrast, untreated samples were not impacted by cell wall composition. At year 5, a strong effect of lignin was highlighted (*r* = −0.93) followed by cellulose (*r* = −0.89) and hemicellulosic carbohydrates (*r* = 0.89), which similarly affected digestion of pretreated miscanthus. Furthermore, compared to the year 4 samples, the three major cell wall polymers, especially cellulose (*r* = −0.74), displayed higher effect on digestion of 5th-year untreated miscanthus. Finally, strong correlations were established between hemicellulosic carbohydrates (*r* = 0.81), lignin (*r* = −0.83), LCI (*r* = −0.85), and enzymatic hydrolysis of untreated miscanthus at year 7. Glucose release from pretreated samples was also strongly impacted by hemicellulosic carbohydrates (*r* = 0.94), lignin (*r* = −0.91), and Hemicellulosic carbohydrates/Cellulose (*r* = 0.94), Lignin/Cellulose (*r* = −0.86), and LCI (*r* = −0.90) ratios. Cellulose conversion after hot water treatment was also positively affected by cell wall extractives on year 4 (*r* = 0.74), year 5 (*r* = 0.84), and year 7 (*r* = 0.73) while untreated genotypes were only slightly affected in year 5.

### Cellulose conversion of the five genotypes at the pilot scale

**Figure 5:**
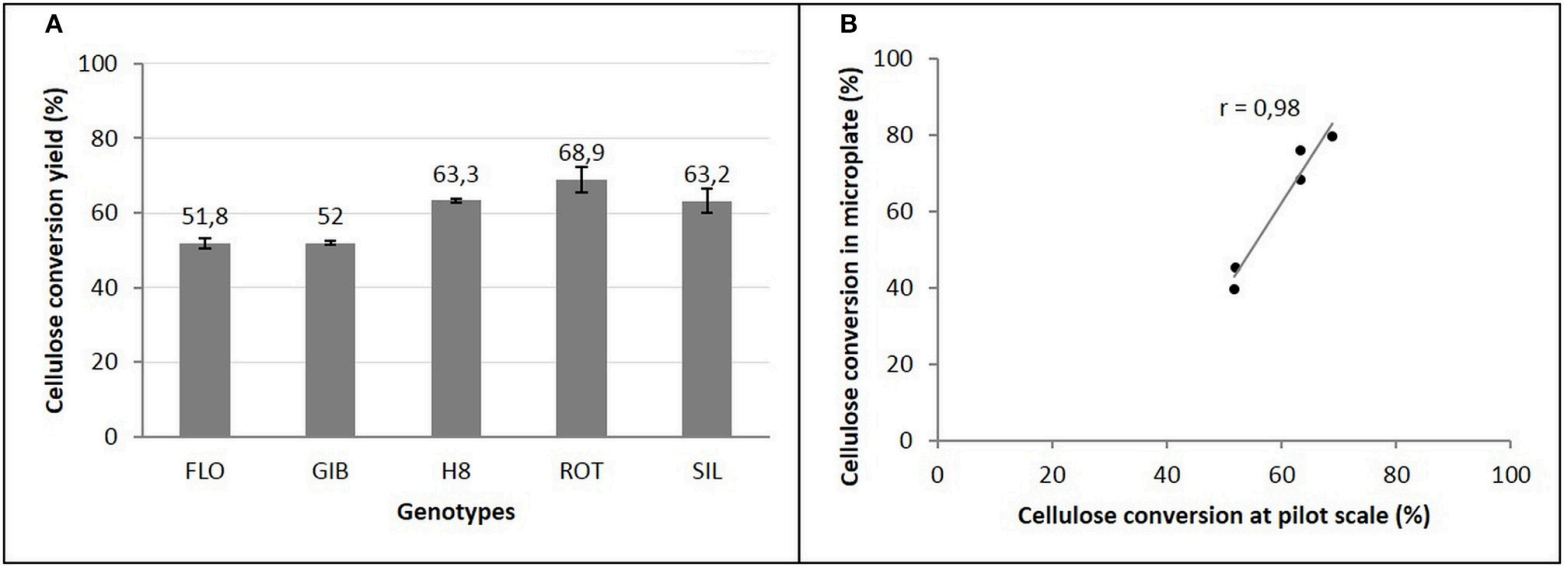
**Cellulose conversion yields of miscanthus genotypes at pilot scale (A)** and its correlation with microplate saccharification **(B)**.

The enzymatic conversion yields of cellulose of steam exploded 7th-year miscanthus at the pilot-scale indicated that ROT genotype is the most digestible (68%), in contrast to H8 and SIL genotypes that express the same cellulose conversion yields (63%), and the less hydrolyzed ones, which are the FLO and GIB genotypes that reach only 52% (Figure 5A).

The comparison of these cellulose conversion yields, to those obtained using our miniaturized assay, revealed a very strong and positive correlation (Figure 5B). Indeed, compared to FLO and GIB, a coefficient of correlation *r* = 0.98 was obtained, confirming the better digestibility of the group formed by H8, ROT, and SIL genotypes.

## Discussion

This study explored the saccharification potential of miscanthus genotypes and year variability effect using pilot and miniaturized assay scales and addressed our following hypothesis: (i) The saccharification potential varied according to the genotype and harvesting year due to variations in the biomass production and in content of the major cell wall components; particularly, cellulose and lignin displayed the same negative effect on cellulose conversion in contrast to hemicelluloses. (ii) The miniaturized assay we developed was reliable for mimicking the saccharification potential of various miscanthus genotypes at the pilot scale. We will, therefore, discuss hereafter the following two points: (i) The relationships between biomass production, content of the main cell wall components, and cellulose conversion according to harvesting year and genotype and (ii) in comparison to pilot scale, the reliability of mimicking the cellulose conversion of various genotypes in miniaturized assay.

### Relationships between biomass production, cell wall composition, and cellulose conversion, according to harvesting year and genotype

Exploring biomass production and composition of the five miscanthus genotypes revealed a large genetic diversity. Based on variance analysis, this biomass quality diversity was found for the following three major cell wall polymers: cellulose, hemicellulosic carbohydrates, and lignin. The cell wall composition reported here is in agreement with previously reported *M*. × *giganteus* and *M. sinensis* data using Van Soest method (Arnoult et al., 2015a). This method allows sequential fractionation of the main cell wall polymers. However, non-cellulosic polysaccharides might be underestimated as neutral detergent solution can solubilize some pectins. In addition the fraction designated as hemicellulosic carbohydrates would thus mostly correspond to heteroxylans with few amounts of xyloglucan, mannan, and pectins (Le Ngoc Huyen et al., 2010; Costa et al., 2016). Furthermore, lignin concentrations as ADL residue were substantially lower than values obtained for miscanthus using Klason method (Le Ngoc Huyen et al., 2010), as previously reported for other grass species (Hatfield et al., 1994; Bertrand et al., 2006). The acid detergent used in the Van Soest method can solubilize acid soluble lignin (Hatfield et al., 1994), giving a lignin-like fraction with a higher degree of recalcitrance as shown in forage digestibility studies (Van Soest, 1963).

Regarding the relationships between biomass production and composition, we highlighted that biomass production was positively correlated to cellulose and lignin, but negatively correlated to hemicellulosic carbohydrates, in agreement with the literature (Xu et al., 2012; Arnoult et al., 2015a). This suggests that miscanthus growth may be accompanied by higher contents in cellulose and lignin while other carbohydrates proportions decrease.

Comparison of released glucose from untreated and hot water-treated genotypes has also revealed that in both conditions, harvesting year and genotypes differentially affect cellulose digestion. Indeed, after pretreatment, harvesting year effect was limited to year 5 (Figure 4), while genotype impact became stronger. In contrast to pretreated genotypes, relationships analysis mainly indicated that cellulose conversion of untreated miscanthus was less impacted by cell wall traits quantified in this present study, suggesting an incomplete and inefficient enzymatic hydrolysis. In fact, biomass production negatively impacted cellulose conversion of hot water-treated samples, but the strength of this relationship was significantly impacted by the two genotype groups and three harvesting years. Similar findings have been recently reported by Domon et al. (2013) while exploring the impact of cold acclimation on miscanthus cell wall composition.

Variations in cellulose conversion yields, according to genotypes and harvesting years, is related to the cell wall and tissue architectures between the less recalcitrant ROT, H8, and SIL genotypes and the more recalcitrant FLO and GIB ones. Indeed, based on our results, the highest cellulose conversion yields displayed by ROT, H8, and SIL can be related to the highest Hemicellulosic carbohydrates/Cellulose ratio and lowest Lignin/Cellulose and LCI ratios, while more recalcitrant FLO and GIB displayed the lowest Hemicellulosic carbohydrates/Cellulose ratio and highest Lignin/Cellulose and LCI ratios. This lignocellulose index (LCI) is reported to be high when biomass is less digestible (Moorhead et al., 2013). Based on correlation analysis, extractives do not seem to explain observed differences on cellulose conversion between the three harvesting years. Hot water pretreatment applied to these genotypes may result in dissolving of hemicelluloses and slight increasing lignin content in the pretreated biomass as reported by Li H.-Q. et al. (2013).

To better understand results highlighted in this study, other cell wall traits must be addressed such as monosaccharide composition of hemicellulose fraction, glycome profile, and cellulose crystallinity index. Indeed, wet chemistry can allow us determining xylan chain substitution by arabinose and other components. For instance, a higher substitution degree of xylan chain by arabinose was reported to positively affect miscanthus saccharification and microbial decomposition (Li F. et al., 2013; Amin et al., 2014). Acetylation of xylan chains has also been reported to create steric hindrance for binding cellulolytic enzymes (Selig et al., 2009; Gille and Pauly, 2012). Phenolic acids, *p*-coumaric and ferulic acid are also known to impact saccharification potential (Belmokhtar et al., 2013). Moreover, the role of minor cell wall components such as xyloglucans, rhamnogalacturonans, and homogalacturonans have been shown to be differentially distributed among different harvests, organs, and genotypes (Costa et al., 2016).

Variations in leaf/stem ratio can also explain differences in cellulose conversion between more and less recalcitrant genotypes as leaves have been reported to be more reactive to cellulases conversion as compared to stems (Le Ngoc Huyen et al., 2010; Costa et al., 2016). From the study of Arnoult et al. (2015a), ROT, H8, and SIL, displaying the highest cellulose conversion yields, show higher leaf/stem ratios (varied from 0.11 to 0.33 for the harvest in February of years 4 and 5) than FLO and GIB, displaying the lowest cellulose conversion yields (leaf stem ratios varied from 0.01 to 0.08 for the harvest in February of years 4 and 5). Indeed, ROT, H8, and SIL genotypes displayed more leaves at the February harvest than FLO and GIB, for which, the major part of the leaves fell to the ground before the harvest. As leaves contain less lignin than stems (Arnoult et al., 2015a), the higher quantity of leaves at harvest can be explained, in that ROT, H8, and SIL genotypes were less recalcitrant and then gave higher cellulose conversion yields. Indeed, the released glucose after enzymatic hydrolysis of hot water-treated miscanthus genotypes was positively correlated to the leaf/stem ratio (*r*-values of 0.57 and 0.79, respectively for the years 4 and 5). The correlation was also positive for the untreated miscanthus genotypes, even if this correlation was lower than for hot water-treated genotypes (*r*-values of 0.03 and 0.66, respectively for the years 4 and 5). The differences in cell wall and tissue architectures, between more and less recalcitrant genotypes, confirmed how hemicelluloses' interaction with cellulose and reduced lignin content was important for achieving higher cellulose conversion yields.

Cellulose conversion inhibition by high lignin and cellulose contents has been reported to be due to unproductive fixation of cellulolytic enzymes on lignin, while hemicelluloses are reported to deposit into cell walls via crosslink to cellulose by hydrogen bonds; thus, reducing cellulose crystallization (Xu et al., 2012; De Souza et al., 2015; van der Weijde et al., 2016). Indeed, reduction in cellulose crystallinity may result in efficient cellulase access to cellulose substrate (Yoshida et al., 2008). Lignin is also assumed to interact with hemicelluloses rather than with cellulose, which can reduce interactions between hemicelluloses and cellulose, thus increasing cellulose crystallinity.

To conclude, the genetic diversity on biomass production and cell wall composition can be used in breeding programs to improve the saccharification potential of genotypes for the objective of bioethanol production.

### Reliability of mimicking the cellulose conversion of various miscanthus genotypes in the miniaturized assay in comparison to the pilot scale

We investigated, for the first time at the pilot scale, the performance of five miscanthus genotypes contrasted for their biomass composition belonging to the species *M*. × *giganteus* (FLO, GIB, and H8) and *M. sinensis* (ROT and SIL). Steam explosion and enzymatic hydrolysis were carried out at the pilot scale in close agreement to previously reported data of other feedstocks (Schell et al., 2003; Thomsen et al., 2006; Rocha et al., 2012). Our results highlighted significant differences between the explored genotypes exhibiting distinct recalcitrance. Compared to the results shown by Schell et al. on corn stover, these results seem to be much lower, probably due to the intrinsic properties of miscanthus (Schell et al., 2003).

Furthermore, we performed hot water pretreatment and enzymatic hydrolysis with saturating amounts of cellulolytic enzymes to screen more samples for their digestibility based on the method recently described by Selig et al. (2010). A high-throughput screening method was initially developed at NREL (Golden, CO, USA) where the pretreatment step used a strong pilot-scale boiler to produce steam heated to 180°C. Here, we adapted this pretreatment step to a laboratory scale using a small steam generator provided from CellKraft® (Sweden). This resulted in a little more time for reaching target temperature but was able to maintain it during 40 min. Results accuracy and reliability have been assessed using 5 normalization controls for inter-plate and inter-assay comparisons. It consists on running a standard biomass in 4 wells in addition to a biomass control well not containing cellulolytic enzymes. During the current study, CV corresponding for these normalization controls was lesser than 1.5% (Supplementary Image 2). The advantage of our high-throughput method therefore, relies in its laboratory scale and reliability.

We highlighted a strong correlation between the pilot and miniaturized assay for cellulose conversion, displaying different patterns between the digestible H8, ROT, and SIL group and the more recalcitrant FLO and GIB group. However, cellulose conversion yields were relatively high according to the miscanthus genotype. This difference between the pilot and miniaturized assays can be explained by the different experimental conditions between the pilot and miniaturized assay, such as pretreatment technology and enzyme loading. Indeed, the better digestibility observed at the pilot scale for the FLO and GIB genotypes may be explained by the presence of sulfuric acid and fiber explosion during the pretreatment carried out at the pilot-scale; while the hot water treatment used in the miniaturized assay seemed less efficient in these recalcitrant biomasses. In contrast, the more digestible genotypes ROT, H8, and SIL responded better to the hot water treatment used in the miniaturized assay. Furthermore, this original approach allowed us to highlight the same trend in the cellulose conversion potential of the five genotypes regardless of the harvesting year. Moreover, the inter-year comparison revealed distinct digestibility, which confirms the importance of our approach based on using sub-optimal pretreatments, while giving high cellulose conversion yields.

Therefore, this high-throughput miniaturized and automated method accurately mimics the performance at the pilot scale and will be reliable for assessing the performance of miscanthus tested in small field plot trials.

## Conclusions

The miniaturized and automated pretreatment and saccharification method we developed allowed us to highlight the variability in saccharification potential according to miscanthus genotypes belonging to the most-studied species *M*. × *giganteus* and *M. sinensis* and harvesting year. This variability in saccharification potential was explained by variations in biomass production and cell wall composition. Indeed, we revealed that changes in the biomass production were positively correlated to cellulose and lignin content and negatively correlated to hemicellulosic carbohydrates polymers. Moreover, the relationship analysis between released glucose after pretreatment and saccharification and biomass quality traits indicated the same strong negative effect of lignin and cellulose, while hemicellulosic carbohydrates significantly increased miscanthus digestibility. For future investigations, it would be interesting to more deeply explore the involvement of esterified and etherified phenolic acids and the arabinose ramification of xylan chains in cell wall cross-linking, to better understand the observed genetic diversity highlighted in this study, especially the positive effect of hemicellulosic carbohydrates.

The results obtained in this study have also shown that our lignocellulosic biomass assessment system, which was developed based on very small quantities of matter in the image of the NREL system (USA), allows a fair and efficient evaluation of the saccharification potential, which has proven to be well correlated with the results obtained at the pilot scale close to industrial reality.

These findings could be used in breeding programs to develop less recalcitrant genotypes for cellulosic ethanol production at the industrial scale since our results at the miniaturized and pilot scales are well-correlated.

## Author contributions

NB developed the miniaturized test and performed the corresponding analyses. SA analyzed the data from the pilot tests. BC contributed to the development of the initial miniaturized test and to the analysis of the enzymatic hydrolysis of cellulose. JPC coordinated the corresponding project and he supervised the development of the miniaturized tests. MB contributed to the development of the initial project. NB and SA wrote the first draft of the manuscript and all authors contributed to the final version.

### Conflict of interest statement

The authors declare that the research was conducted in the absence of any commercial or financial relationships that could be construed as a potential conflict of interest.

## Supplementary material

Supplementary Image 1 **Subdivision of genotypes into two distinct groups based on biomass traits**. Biomass yield **(A)**, Cellulose **(B)**, Hemicellulosic carbohydrates **(C)**, Lignin **(D)**, Hemicellulosic carbohydrates/Cellulose **(E)**, Lignin/Cellulose **(F)**, LCI **(G)**.

Supplementary Image 2 **Repeatability of the saccharification assay**.

## Abbreviations

ADF: acid detergent fiber
ADL: acid detergent lignin
CV: coefficient of variation
DM: dry matter
ha: hectare
HPAEC: high-performance anion-exchange chromatography
HWT: hot water pretreatment
LCI: lignocellulose index
NDF: neutral detergent fiber
NDS: neutral detergent soluble
t: ton
UT: untreated
PCR: polymerase chain reaction.
