# Supplementary figures and images for: Saccharification Performances of Miscanthus at the Pilot and Miniaturized Assay Scales: Genotype and Year Variabilities According to the Biomass Composition
Source: Front Plant Sci. 2017 May 29;8:740. doi: 10.3389/fpls.2017.00740 (PMC5447034; doi:10.3389/fpls.2017.00740)

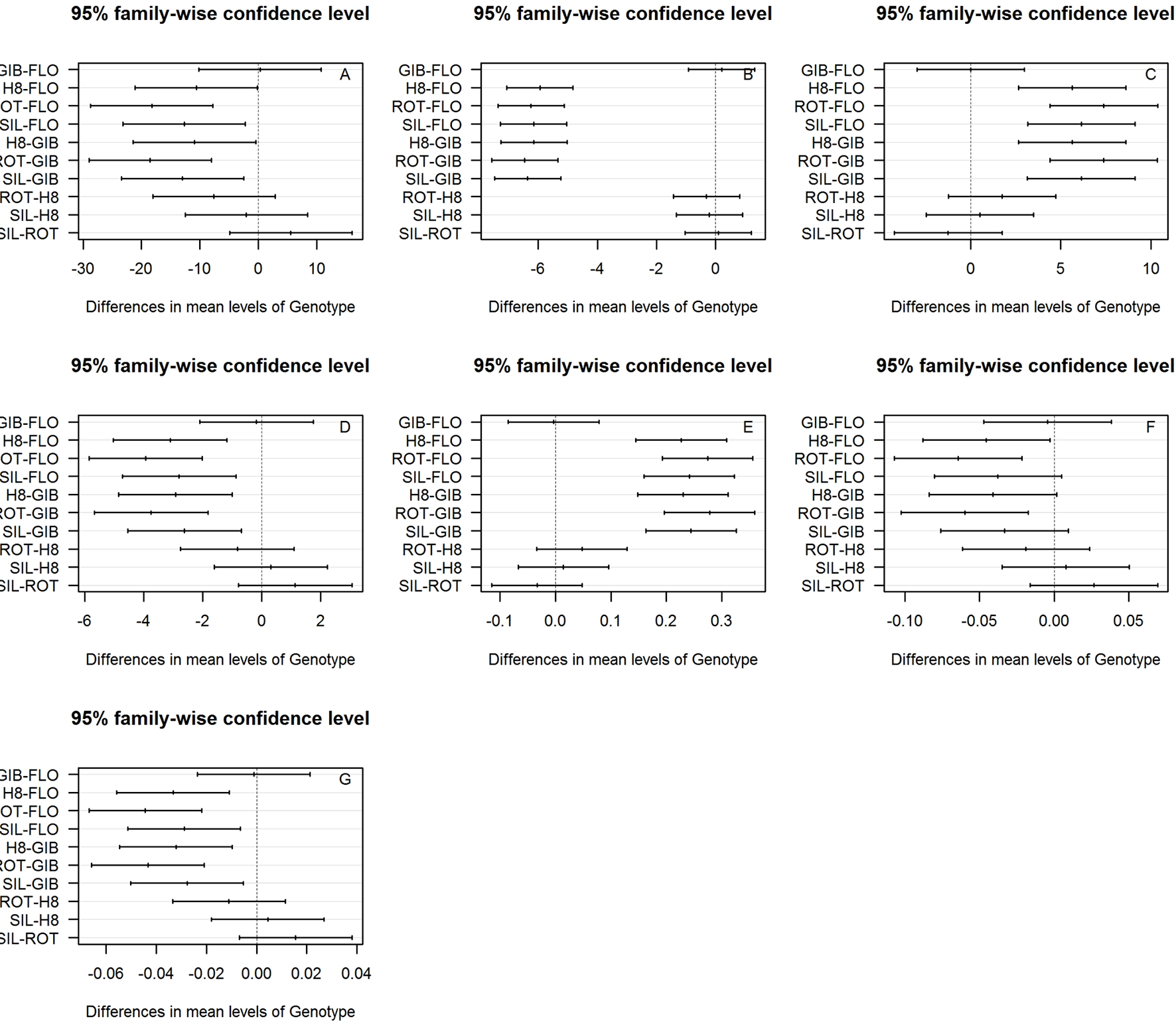

Supplement: Supplementary Image 1 — Subdivision of genotypes into two distinct groups based on biomass traits. Biomass yield (A), Cellulose (B), Hemicellulosic carbohydrates (C), Lignin (D), Hemicellulosic carbohydrates/Cellulose (E), Lignin/Cellulose (F), LCI (G). [file Image1.tif]

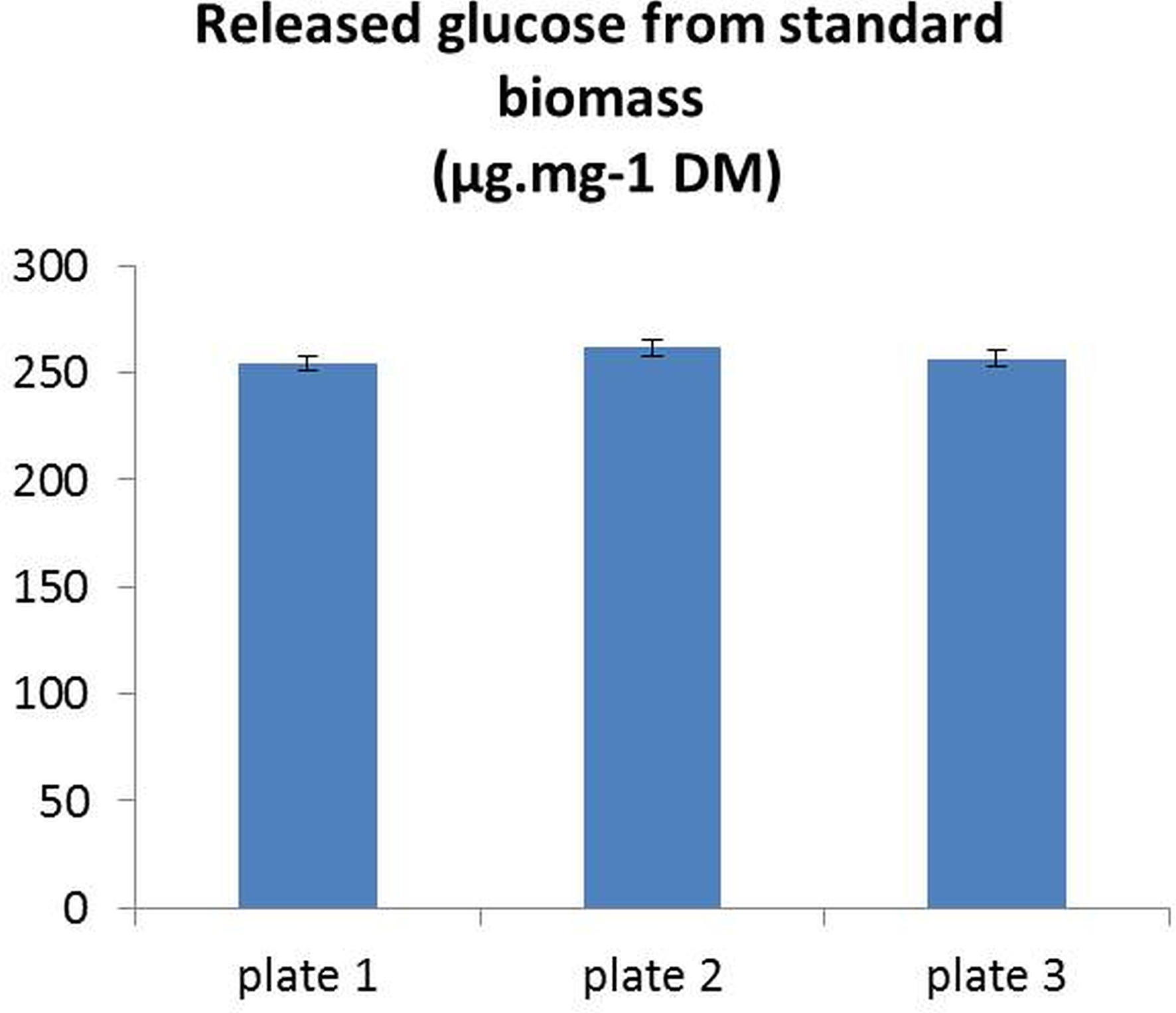

Supplement: Supplementary Image 2 — Repeatability of the saccharification assay. [file Image2.TIF]
